# Correction: How does symbolic success affect redistribution in left-wing voters? A focus on the 2017 French presidential election

**DOI:** 10.1371/journal.pone.0233868

**Published:** 2020-05-21

**Authors:** The PLOS ONE Staff

The Funding statement is incorrect. The publisher apologizes for the error. The correct Funding statement is as follows: The publication of this study was funded by Agence nationale de la recherche (ANR) "Coping with Heterogeneous Opinions–CHOp" [ANR-17-CE26-0003]. The funder of this study had no additional role in study design, data collection and analysis, decision to publish, or preparation of the manuscript. No additional external funding was received for this study
