# Supplementary material for: How does symbolic success affect redistribution in left-wing voters? A focus on the 2017 French presidential election
Source: PLoS One. 2020 Mar 16;15(3):e0229096. doi: 10.1371/journal.pone.0229096 (PMC7075674; doi:10.1371/journal.pone.0229096)
Supplement: S1 Appendix — (DOCX) [file pone.0229096.s001.docx]

**Appendix A. ANOVA table for redistributive behavior in the disinterested dictator game including all groups of voters.**

|  | *η*_p_ ^2^ | S.S. | d.f. | F | p |
| --- | --- | --- | --- | --- | --- |
| Gender | 0.0113 | 1074 | 1 | 7.006 | 0.008 |
| Age | 0.0039 | 367 | 1 | 2.396 | 0.122 |
| Status | 0.0006 | 57 | 1 | 0.373 | 0.542 |
| Vote1 | 0.0683 | 6878 | 5 | 8.971 | <0.001 |
| Status:Vote1 | 0.0138 | 1313 | 5 | 1.713 | 0.130 |
| Residuals |  | 93848 | 612 |  |  |

*Note.* As some categories of First-round vote have very few observations (e.g. Arthaud, Poutou, Lassalle, Cheminade, Asselineau, Dupont-Aignan), they were tentatively merged according to political categories, as follows: 1 Arthaud and 15 Poutou voters were merged with Mélenchon voters (N = 235); 2 Lassalle voters were merged with Macron voters (N = 168); 2 Cheminade voters were merged with Fillon voters (N = 36); 5 Asselineau, 6 Dupont-Aignan, and 8 Le Pen votes were merged in the “FarRight” category (N = 19); and finally 18 Blank votes and 29 abstentions were merged in the “NoVote” category (N = 47).
