# Supplementary material for: How does symbolic success affect redistribution in left-wing voters? A focus on the 2017 French presidential election
Source: PLoS One. 2020 Mar 16;15(3):e0229096. doi: 10.1371/journal.pone.0229096 (PMC7075674; doi:10.1371/journal.pone.0229096)
Supplement: S2 Appendix — (DOCX) [file pone.0229096.s002.docx]

**Appendix B. Instructions (Screen Shot and Translation).**

B.1 General Instructions


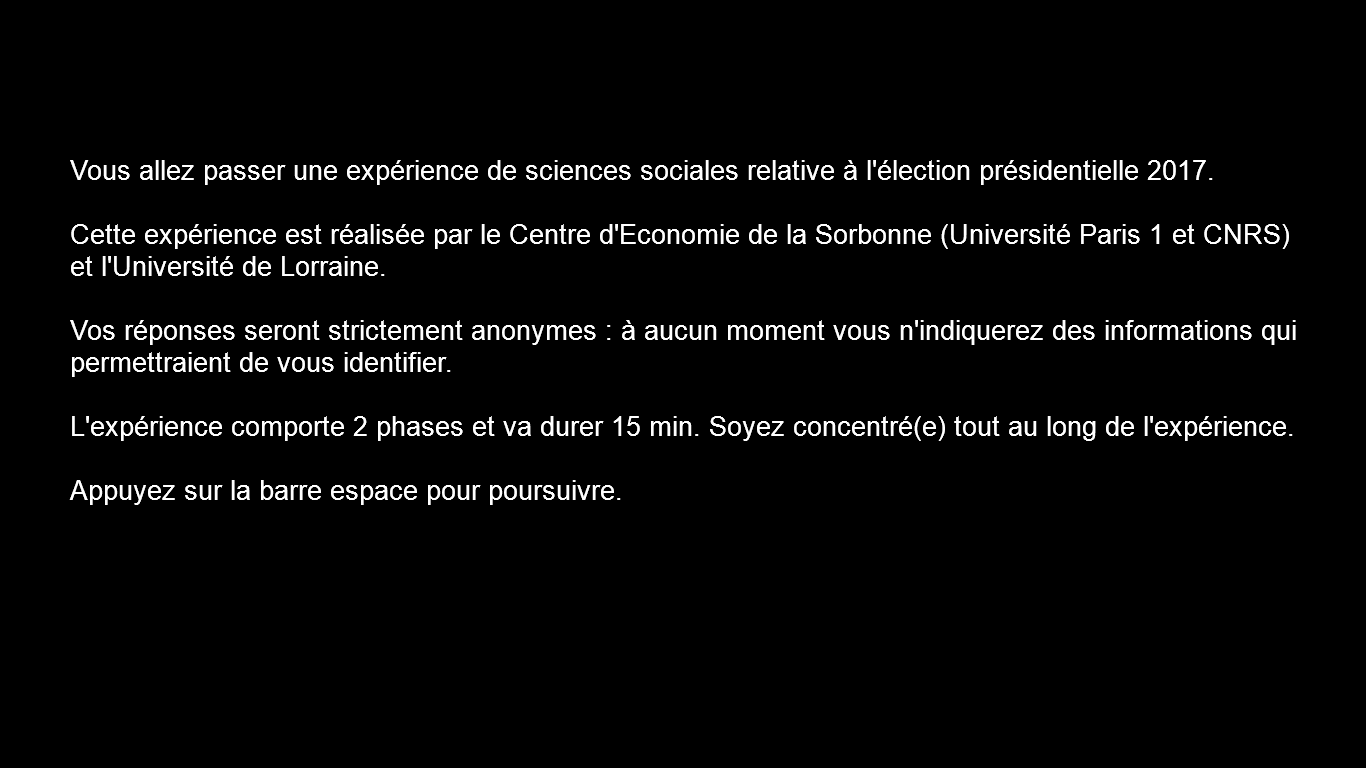


Translation: *“You are about to take part to a social science experiment on the 2017 French presidential election. This experiment is conducted by the Centre d’Économie de la Sorbonne (Université Paris 1 and CNRS) and the Université de Lorraine. Your responses are totally anonymous: You will not reveal information that would identify you. The experiment is divided into two stages and takes 15 min. Stay focused during the experiment. Press the spacebar to continue.”*

B.2 Instructions in the disinterested dictator game


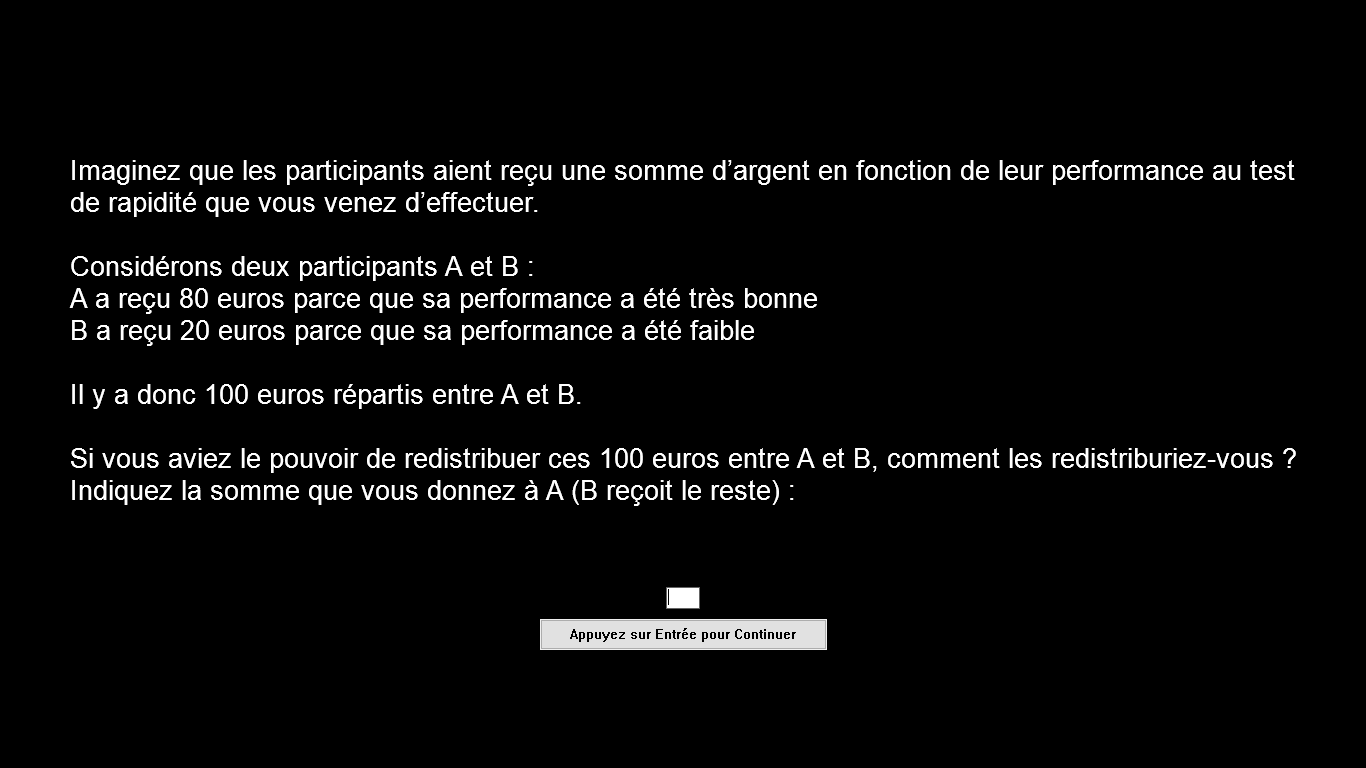


Translation: *“Imagine that the participants received an amount of money based on their performance on the speed test you just performed.*

*Consider two participants A and B:
A received 80 euros because his performance was very good
B received 20 euros because his performance was weak*

*There is therefore 100 euros to split between A and B.*

*If you had the power to redistribute the 100 euros between A and B, how do you redistribute them? Indicate the amount you would give to A (B receiving the rest)”*
