# Supplementary material for: How does symbolic success affect redistribution in left-wing voters? A focus on the 2017 French presidential election
Source: PLoS One. 2020 Mar 16;15(3):e0229096. doi: 10.1371/journal.pone.0229096 (PMC7075674; doi:10.1371/journal.pone.0229096)
Supplement: Attachment — Submitted filename: Rebuttal letter.docx [file pone.0229096.s003.docx]

*Comment 1: mismatch between self-reported votes and actual votes*

The initial evaluation of the manuscript mentioned:

“In this case, you have presented self-reported voting intention as actual voting behavior without verification, and have included assumptions around the relationship between self-reported voting behavior and political views without including data supporting this assumption. In light of these concerns, we felt the interpretation of your results were not supported by the data and that your results are better stated as hypotheses instead of conclusions.”

In addition, the email offering us to provide a revision, mentioned:

“the distinction between self-reported and actual voter behavior has important implications for the data and its interpretation give the discrepancies between reported and actual voter behavior described in recent elections including the election under study in this manuscript, and we would require that this and the associated limitations be addressed in the revised manuscript.”

First, we would like to thank the editor(s) for bringing our attention to this issue and for the opportunity to clarify and correct our presentation and interpretation of the data in the revised manuscript.

We believe that this point is indeed important to acknowledge, as polls (i.e. self-reported votes) and actual votes can indeed differ, as famously illustrated in, e.g., the 2016 US presidential election, the 2016 “Brexit” referendum, or the 2002 French presidential election. However, we note that in the case of the election under study here (2017 French presidential election), the last polls were accurate, as can be seen in the table below which includes the last 6 polls for this election, conducted by major polling institutes:

| **Polling firm** | **Fieldwork date** | **Sample size** | **Abs.** | [**Arthaud**](https://en.wikipedia.org/wiki/Nathalie_Arthaud) [**LO**](https://en.wikipedia.org/wiki/Lutte_Ouvri%C3%A8re) | [**Poutou**](https://en.wikipedia.org/wiki/Philippe_Poutou) [**NPA**](https://en.wikipedia.org/wiki/New_Anticapitalist_Party) | [**Mélenchon**](https://en.wikipedia.org/wiki/Jean-Luc_M%C3%A9lenchon) [**FI**](https://en.wikipedia.org/wiki/La_France_Insoumise) | [**Hamon**](https://en.wikipedia.org/wiki/Beno%C3%AEt_Hamon) [**PS**](https://en.wikipedia.org/wiki/Socialist_Party_(France)) | [**Macron**](https://en.wikipedia.org/wiki/Emmanuel_Macron) [**EM**](https://en.wikipedia.org/wiki/En_Marche!) | [**Lassalle**](https://en.wikipedia.org/wiki/Jean_Lassalle) [**Résistons!**](https://en.wikipedia.org/wiki/R%C3%A9sistons!) | [**Fillon**](https://en.wikipedia.org/wiki/Fran%C3%A7ois_Fillon) [**LR**](https://en.wikipedia.org/wiki/The_Republicans_(France)) | [**Dupont-Aignan**](https://en.wikipedia.org/wiki/Nicolas_Dupont-Aignan) [**DLF**](https://en.wikipedia.org/wiki/Debout_la_France) | [**Asselineau**](https://en.wikipedia.org/wiki/Fran%C3%A7ois_Asselineau) [**UPR**](https://en.wikipedia.org/wiki/Popular_Republican_Union_(2007)) | [**Le Pen**](https://en.wikipedia.org/wiki/Marine_Le_Pen) [**FN**](https://en.wikipedia.org/wiki/National_Front_(France)) | [**Cheminade**](https://en.wikipedia.org/wiki/Jacques_Cheminade) [**S&P**](https://en.wikipedia.org/wiki/Solidarity_and_Progress) |
| --- | --- | --- | --- | --- | --- | --- | --- | --- | --- | --- | --- | --- | --- | --- |
|  |  |  |  |  |  |  |  |  |  |  |  |  |  |  |
| [2017 election](https://en.wikipedia.org/wiki/2017_French_presidential_election) | 23 Apr 2017 | – | 22.23% | 0.64% | 1.09% | 19.58% | 6.36% | **24.01%** | 1.21% | 20.01% | 4.70% | 0.92% | **21.30%** | 0.18% |
| [Odoxa](http://www.odoxa.fr/wp-content/uploads/2017/04/Intention-de-vote-presidentielle-Odoxa-LePoint_210417.pdf) | 21 Apr 2017 | 666 | – | 0% | 1% | 19% | 7.5% | **24.5%** | 0.5% | 19% | 4.5% | 1% | **23%** | 0% |
| [BVA](https://staticswww.bva-group.com/wp-content/uploads/2017/04/Intentions-de-vote-Vague-19-POP2017-21-avril-2017-Pr%C3%A9sentation.pdf) | 20–21 Apr 2017 | 1,134 | 20% | 0.5% | 1.5% | 19.5% | 8% | **23%** | 1% | 19% | 4% | 0.5% | **23%** | <0.5% |
| [Ifop-Fiducial](http://dataviz.ifop.com:8080/IFOP_ROLLING/IFOP_21-04-2017.pdf) | 18–21 Apr 2017 | 2,823 | 27% | 0.5% | 1.5% | 18.5% | 7% | **24.5%** | 1% | 19.5% | 4% | 1% | **22.5%** | <0.5% |
| [Odoxa](http://www.odoxa.fr/wp-content/uploads/2017/04/Intention-de-vote-presidentielle-Odoxa-LePoint_210417.pdf) | 20 Apr 2017 | 1,433 | – | 0.5% | 1.5% | 19.5% | 6% | **25%** | 1% | 19.5% | 4% | 1% | **22%** | 0% |
| [Elabe](https://elabe.fr/wp-content/uploads/2017/04/20042017_bfmtv_lexpress_intentions-de-vote-presidentielles-vague-10.pdf) | 19–20 Apr 2017 | 1,445 | 29% | 0.5% | 1.5% | 19.5% | 7% | **24%** | 1% | 20% | 4% | 1% | **21.5%** | <0.5% |
| [Ipsos](https://www.ipsos.com/sites/default/files/files-fr-fr/doc_associe/intention_de_vote_ipsos_ft-rf_-_21_avril_2017.pdf) | 19–20 Apr 2017 | 1,401 | 27% | 0.5% | 1.5% | 19% | 7.5% | **24%** | 1.5% | 19% | 4% | 1% | **22%** | <0.5% |

From: <https://en.wikipedia.org/wiki/Opinion_polling_for_the_2017_French_presidential_election>

We note that in practice, studies of voting behavior typically assume that reported votes correspond to actual votes and political views (e.g. Friese et al., 2012, PLOS ONE, Lee et al., 2016, in PLOS ONE; Jacoby, 2010, in Electoral Studies; Payne, 2010, Journal of Experimental Social Psychology; Greenwald et al., 2009, in Analyses of Social Issues and Public Policy). This assumption is so common that it is in fact not even mentioned in most papers. Here, we will make it explicit.

We will acknowledge that participants in our sample might not be immune from biases when reporting their votes. Typically, in French elections, votes towards rightwing or far-right parties are under-estimated. We now also discuss how reported votes in our sample could be biased towards the left. However, we do not see how this bias might explain the interaction between groups and status on redistributive behavior, which constitutes our main result.

To present these elements, we have modified the abstract and the main text of the manuscript to refer to “reported votes” instead of simply “votes” where appropriate, and we have added to the discussion the following paragraph highlighting the possible discrepancy between self-reported votes and actual votes:

“The second limitation relates to the possible discrepancy between actual votes and reported votes in our participants. Poll estimates (based on self-reported votes) and actual votes can indeed differ, as famously illustrated in the 2016 US presidential election, the 2016 “Brexit” referendum, or the 2002 French presidential election, amongst others. However, we note that in the case of the election under study here (2017 French presidential election) the last polls were very accurate. One reason for the discrepancy between self-reported votes and actual votes might be a social desirability bias by which right-wing or far-right votes are expressed less easily and therefore under-estimated in opinion polls (see e.g. Brownback & Novotny, 2018). Critically, polling institutes use adjustment procedures to take into this bias when producing their estimates, but we did not. Therefore, right-wing opinions/votes in our sample might have been under-estimated. In sum, although we followed the common practice in studies of voting behavior, and used the terms “Melenchon voters”, “Hamon voters” or “Macron voters”, one should bear in mind that our data is about self-reported votes, which might have differed from actual votes.”

*Comment 2: mismatch between self-reported votes and actual votes*

Additionally, we felt the concluding portion of your manuscript, lines 257 through 269, contained declarations that were not thoroughly supported by the primary research in this manuscript or referenced literature. In our view, your conclusions stray too far toward subjective opinion to justify inclusion in PLOS ONE.

We thank the editor for this comment. This paragraph was indeed presented as a speculative, and was not essential to our discussion. We have removed it in the revised manuscript.

**References**

Lee et al., 2016, PLOS ONE, Voting Intention and Choices: Are Voters Always Rational and Deliberative? 
<https://journals.plos.org/plosone/article?id=10.1371/journal.pone.0148643>

Jacoby, 2008, Electoral Studies. Policy attitudes, ideology and voting behavior in the 2008 Election. 
<https://www.sciencedirect.com/science/article/pii/S0261379410000429>

Payne, 2010, Journal of Experimental Social Psychology, Implicit and explicit prejudice in the 2008 American presidential election. 
<https://www.sciencedirect.com/science/article/pii/S002210310900273X>

Greenwald et al., 2009, Analyses of Social Issues and Public Policy, Implicit Race Attitudes Predicted Vote in the 2008 U.S. Presidential Election. 
<https://spssi.onlinelibrary.wiley.com/doi/full/10.1111/j.1530-2415.2009.01195.x>

Friese et al, 2012, PLOS ONE. Do Implicit Attitudes Predict Actual  Voting Behavior Particularly for Undecided Voters? 
<https://journals.plos.org/plosone/article?id=10.1371/journal.pone.0044130>
