# Supplementary material for: How does symbolic success affect redistribution in left-wing voters? A focus on the 2017 French presidential election
Source: PLoS One. 2020 Mar 16;15(3):e0229096. doi: 10.1371/journal.pone.0229096 (PMC7075674; doi:10.1371/journal.pone.0229096)
Supplement: Attachment — Submitted filename: Response to Reviewers.docx [file pone.0229096.s004.docx]

Reviewer #1: Referee’s report - Manuscript ID PONE-D-19-26429R3

REFEREE R3: Many thanks for these answers. As I said in my previous reports, I truly believe that this paper investigates a relevant question and I enjoyed reading it. Over this process, I believe that the paper has improved a lot. Yet, there is still something in need of clarification.

As I said in my previous email, it is not good to exclude observations. Therefore, I wonder if it is possible to see (and report in the paper – maybe in the annex) the results of the regression including the observations excluded.

AUTHOR/S R3: Regarding the exclusion of observations, we mentioned that few observations were excluded due to a technical error in the data collection. In the paragraph “Description of our sample”, we added the following sentence:

“(due to a technical error in the data collection, redistribution choices could only be analyzed for 626 participants).”

We ran the regression including all observations and added the results in the Annex (Appendix A). Following the recommendation of the reviewer below, we ran the regression adding only gender and age as covariates. Moreover, as some categories of First-round vote have few observations (e.g. Arthaud, Poutou, Lassalle, Cheminade, Asselineau, Dupont-Aignan), observations were merged as follows: Arthaud and Poutou votes were merged with Mélenchon (N = 235); Lassalle votes were merged with Macron (N = 168); Cheminade votes were merged with Fillon (N = 36); Asselineau, Dupont-Aignan, and Le Pen votes were merged in the “FarRight” category (N = 19); Blank votes and abstentions were merged in the “NoVote” category (N = 47). We added the following sentence in the main text:

“For completeness, we also report in Appendix A the results of a regression over all participants, including those who reported a different First-round vote.”

Appendix A: ANOVA table for redistributive behavior in the disinterested dictator game including all groups of voters.

|  | *η*_p_ ^2^ | S.S. | d.f. | F | p |
| --- | --- | --- | --- | --- | --- |
| Gender | 0.0113 | 1074 | 1 | 7.006 | 0.008 |
| Age | 0.0039 | 367 | 1 | 2.396 | 0.122 |
| Status | 0.0006 | 57 | 1 | 0.373 | 0.542 |
| Vote1 | 0.0683 | 6878 | 5 | 8.971 | <0.001 |
| Status:Vote1 | 0.0138 | 1313 | 5 | 1.713 | 0.130 |
| Residuals |  | 93848 | 612 |  |  |

REFEREE R3: Also, I would be happy to see if participants in the overachiever or underachiever groups are different not only in their socio – demographic characteristics but also on “Vote 1”.

AUTHOR/S: We verified that Vote 1 and Status were not associated, by running an ANOVA with Status as a dependent variable and Vote 1 as an independent variable (F(2,503)=2.06, p=.13). In addition, we conducted separate tests comparing the proportion of votes for Mélenchon, Hamon and Macron between overachievers and underachievers. These tests indicated no difference between groups. This information about Vote 1 is now included in the table (Table 2).

| Condition | N | Age (SD) | Gender  (% women) | First-round vote  Mélenchon / Hamon / Macron |
| --- | --- | --- | --- | --- |
| Overachiever | 248 | 33.73 (15.34) | 0.54 | 0.41 / 0.28 / 0.31 |
| Underachiever | 258 | 33.28 (14.06) | 0.60 | 0.46 / 0.20 / 0.34 |
| test |  | t = 0.344 | χ² = 1.64 | χ² = 1.10 / 3.67 / 0.29 |
| p |  | 0.731 | 0.2 | 0.29 / 0.06 / 0.59 |

REFEREE R3: Reading again the paper, I have also some additional suggestion/recommendation. I think that the paper would improve if all the results discussed in the paper are also summarized using tables. It would be great to have a table summarizing the results discussed between the line 191 and 193.

AUTHOR/S: We replaced Figure 3 by a new table (Table 4) indicating means and standard errors of the amount of money allocated to A in the Disinterested Dictator Game as a function of First-round vote and Status.

Table 4
*Means and standard errors of the amount of money allocated to A in the Disinterested Dictator Game as a function of First-round vote and Status.*

| First-round vote  Status | Mélenchon | Hamon | Macron |
| --- | --- | --- | --- |
| Overachiever | 60.5 (1.28) | 59.2 (1.30) | 64.4 (1.58) |
| Underachiever | 56.7 (0.91) | 62.3 (1.86) | 63.0 (1.33) |

REFEREE R3: By contrast, I think that the results reported in Table 4 could not be considered valid. Some of the controls might have been affected by the manipulation e.g. fatalism and views on income inequality. Therefore, the inclusion of these variables as controls may introduce some bias in the estimation. In spite of that, I would suggest to replicate the baseline estimation adding only gender and age as controls.

AUTHOR/S: We followed the recommendation of the reviewer and we now report the regression analysis adding only gender and age as covariates (Table 5). The results were virtually unchanged.

Table 5

*ANOVA table for redistributive behavior in the disinterested dictator game. The different factors included in the model are the effects of Gender, Age, Status in the experiment (Overachiever vs. Underachiever), First-round vote and the interaction between Status and First-round vote.*

|  | *η*_p_ ^2^ | S.S. | d.f. | F | p |
| --- | --- | --- | --- | --- | --- |
| Gender | 0.0080 | 584 | 1 | 4.001 | .046 |
| Age | 0.0051 | 373 | 1 | 2.553 | .111 |
| Status | 0.0026 | 188 | 1 | 1.291 | .256 |
| Vote1 | 0.0239 | 1781 | 2 | 6.101 | .002 |
| Status:Vote1 | 0.0119 | 874 | 2 | 2.992 | .051 |
| Residuals |  | 72701 | 498 |  |  |

We have re-written the paragraph accordingly:

“Noteworthy, to evaluate whether our results were robust to changes in model specification, we conducted a new regression analysis, in which we added gender and age as covariates (Table 5). This regression revealed that gender affected redistribution, with women redistributing more than men, replicating previous findings (e.g. Capraro, 2019; Corneo & Grüner, 2002). This analysis also indicated a main effect of the First-round vote, and confirmed the interaction between Status and First-round vote. When examining the effect of Status separately for the 3 groups of voters, again adding gender and age as covariates, we found that redistributive behavior was affected by Status only for Mélenchon voters (*F*(1, 215) = 5.54, *p* = .020, *η*_p_ ^2^ = .0251), but not for Hamon voters (*F*(1, 117) = 2.09, *p* = .151, *η*_p_ ^2^ = .0176) or Macron voters (*F*(1, 162) = 0.454, *p* = .502, *η*_p_ ^2^ = .0028) replicating our main finding. For completeness, we also report in Appendix A the results of a regression over all participants, including those who reported a different First-round vote.”

REFEREE R3: Finally, I think that the paper would benefit for a better explanation of the variable used in the analysis. How is the First-round vote variable used in the regression is defined after the exclusion of some observations?

AUTHOR/S:

In the paragraph “Description of our sample”, we have made more explicit the fact that in our analysis, the categories of the First-round vote variable are restricted to Mélenchon, Hamon, and Macron. We added the following sentence:

“Accordingly, in what follows the variable “First-round vote” is a categorical variable with 3 possible values, namely Mélenchon, Hamon, and Macron.”
