# Supplementary material for: How does symbolic success affect redistribution in left-wing voters? A focus on the 2017 French presidential election
Source: PLoS One. 2020 Mar 16;15(3):e0229096. doi: 10.1371/journal.pone.0229096 (PMC7075674; doi:10.1371/journal.pone.0229096)
Supplement: Attachment — Submitted filename: Response to Reviewers.docx [file pone.0229096.s005.docx]

Editor’s letter :

I have now collected one review from one expert in the field. Unfortunately, I was unable to find a second reviewer. However, since this review is very detailed and thorough, I have opted for making a decision on your manuscript with only one review. As you will see, the reviewer is quite positive, but has several major comments. Therefore, I would like to invite you to revise your manuscript according to the reviewer's comment. Additionally, after reading your manuscript, I would like to add one more comment. I have myself done a lot of work on redistributive behavior, which I think is quite relevant to your discussion regarding the determinants of redistributive behavior, so you might want to have a look at it (of course, citing this work is not a requirement). In Capraro & Rand (2018) and Tappin & Capraro (2018) we found that redistributive behavior in the trade-off game is driven by moral preferences for doing the right thing and, consequently, it very much depends on how the decision problem is framed; in Capraro (2019), I found that women are more likely than men to choose an equitable distribution over an efficient one.

Looking forward for the revision.

References

Capraro V (2019) Gender differences in the equity-efficiency trade-off. Available at <https://ssrn.com/abstract=3386124>.

Capraro V, Rand DG (2018) Do the right thing: Experimental evidence that preferences for moral behavior, rather than equity and efficiency per se, drive human prosociality. Judgment and Decision Making 13, 99-111.

Tappin BM, Capraro V (2018) Doing good vs. avoiding bad in prosocial choice: A refined test and extension of the morality preference hypothesis. Journal of Experimental Social Psychology 79, 64-70.

We thank you for bringing this work to our attention. We now refer to Capraro & Rand (2018) in our introduction. In addition, we now include gender as a covariate in our regression analysis. We have found indeed that women redistributed more than men, but this did not change our main results. Regarding this gender effect, we refer to Capraro (2019).

We now report our regression analysis as follows:

“Noteworthy, to evaluate whether our results were robust to changes in model specification, we conducted a new regression analysis, in which we added gender, age and the 5 self-report measures as covariates. This regression revealed that fatalism, views on income equality, and economic patriotism were significant predictors of redistributive behaviors. In addition, we found that gender affected redistribution, with women redistributing more than men, replicating previous findings (e.g. Capraro, 2019; Corneo & Grüner, 2002). We note that the main effect of the First-round vote did not reach significance in this new analysis, but critically the interaction between Status and First-round vote remained significant (Table 2). When examining the effect of Status separately for the 3 groups of voters, again adding the 5 self-report measures as covariates, we found that redistributive behavior was affected by Status only for Mélenchon voters (*F*(1, 210) = 6.03, *p* = .0149, *η*_p_ ^2^ = .0279), but not for Hamon voters (*F*(1, 112) = 2.78, *p* = .098, *η*_p_ ^2^ = .0242) or Macron voters (*F*(1, 157) = .790, *p* = .376, *η*_p_ ^2^ = .0050). replicating our main finding.”

We have also modified Table 2 accordingly. The new Table 2 is as follows:

*ANOVA table for redistributive behavior in the disinterested dictator game. The different factors included in the model are the effects of Gender, Age, self-report measures (attitude towards Income inequality, Economic patriotism, Fatalism, Attitude towards France, Political position), Status in the experiment (Overachiever vs. Underachiever), First-round vote and the interaction between Status and First-round vote.*

|  | *η*_p_ ^2^ | S.S. | d.f. | F | p |  |
| --- | --- | --- | --- | --- | --- | --- |
| Gender | 0.0097 | 628 | 1 | 4.81 | .029 | * |
| Age | 0.0006 | 36 | 1 | 0.28 | .598 |  |
| Income inequality | 0.0135 | 879 | 1 | 6.73 | .010 | ** |
| Economic patriotism | 0.0139 | 906 | 1 | 6.94 | .009 | ** |
| Fatalism | 0.0482 | 3260 | 1 | 24.97 | <.001 | *** |
| Attitude France | 0.0030 | 193 | 1 | 1.48 | .225 |  |
| Political position | 0.0018 | 119 | 1 | 0.91 | .340 |  |
| Status | 0.0029 | 187 | 1 | 1.43 | .232 |  |
| Vote1 | 0.0066 | 425 | 2 | 1.63 | .197 |  |
| Status:Vote1 | 0.0148 | 967 | 2 | 3.70 | .025 | * |
| Residuals |  | 64360 | 493 |  |  |  |

Reviewer #1: Referee’s report - Manuscript ID PONE-D-19-26429

This paper uses experimental data to examine how redistributive preferences are shaped by the experience of success and political views. This experiment was implemented during the recent presidential election in France. It finds that redistributive behavior is affected primarily by political opinions e.g. Mélenchon voters redistribute more than other groups. In addition, the paper shows that redistributive behavior is influenced by the exogenous manipulation of experiences of success. Interestingly, this effect is not homogenous across political groups and only the Mélenchon voters were significantly affected by the status.

The paper is well-written and competently conducted. The literature review is fine although I would suggest referring also to the literature investigating experimentally if and how information about actual inequality (and political position) affects policy preferences. Overall, this paper investigates a relevant question and I enjoyed reading it.

We thank the reviewer for these positive comments.

Regarding the comment about the experimental literature, we have now expended the third paragraph of introduction to include some references showing that providing information about actual inequality affects support for redistribution. The paragraph now reads as follows:

“Surveys have shown that such beliefs about the determinants of inequality are not homogeneous across the population (e.g. Cozzarelli, Wilkinson, Tagler, 2001). Relatedly, support for redistributive policies varies across social groups defined by race, gender, age or socioeconomic status (Keely & Tan, 2008). In the United States, whites are more averse to redistribution than blacks, even after controlling for individual characteristics such as income, education, etc. (e.g., Gilens, 1999; Alesina & La Ferrara, 2005). Past upward mobility also decreases the support for redistribution (e.g. Guillaud & Sauger, 2013; Alesina & La Ferrara, 2005). Some of these observations have been confirmed by experimental data. For instance, when participants are presented with mock news articles reporting high (vs. low) rates of social mobility, their tolerance for inequality increased (Shaffir, Wiwad, Aknin, 2016). Providing American adults with factual information about the rise of inequalities in the United States (vs. control information) increased their beliefs that economic inequalities are due to structural rather than individual factors and increased support for redistribution (McCall et al., 2017; Boudreau & MacKenzie, 2018).”

Despite that, I am not sure about the results. I have a number of concerns and I turn to discuss each of these issues in the remainder of this report.

First, the sample is heavily biased to left position and is far to be representative of the overall population. Although, I appreciate that authors are well aware about this limitation, I still think that this is a big issue. The worry is that one would expect the left-wing participants to possibly have quite different preferences from the general population. How much we can take from this work and extend to other groups or even other contexts? Moreover, we have also to take in consideration the timing of the survey. Indeed, voters usually receive more information in the period of elections and may react to them more strongly - at least in the short term.

We are indeed aware of this limitation due to the bias in our sample, which was already acknowledged in the discussion. In the revised manuscript, we have made more explicit the possibility that right-wing or far-right voters could have exhibited a different behavior.

We now also highlight that the timing of the survey is an important part of the specific context of our study. Generalization to other populations and other contexts is always an issue in experimental studies. However, we believe that since it is an empirical issue, it can be tackled in future work. We have added the following paragraph in the discussion section to address this point:

“In addition, one could argue that a second limitation of the present work is related to the specific timing of the study, which took place during the French presidential election. This specific timing was chosen on purpose for two reasons. One reason was to benefit from the increased interest towards political topics at this time. The other reason was to probe voters’ redistributive behavior at a time that constitutes an important step in the democratic process. However, we acknowledge that it is possible that voters’ behavior in our study is unusual, because of this unusual timing. Voters may receive more information in the context of an election, and they may react more strongly to information delivered in this context. Whether our results would generalize to another context unrelated to a particular election thus remains an open empirical issue.”

Second, there are not enough information about groups characteristics. Are participants in the overachiever or underachiever groups different in their characteristics? Did the exclusions of some observations affect the balancing properties of the sample? How much people voting Hamon are different from those voting Macron, for example? These are very important information to understand the validity of the final results.

We thank the reviewer for this comment. We have clarified in the discussion that group characteristics did not differ between Mélenchon voters and Hamon voters (Macron voters were older and more male on average), and between overachievers and underachievers.

“Explanations of this finding in terms of age, sex, or socio-economic status are unlikely in our dataset as Mélenchon voters and Hamon voters did not differ significantly on these variables (Table 3). In addition, we verified that our Status manipulation was truly random with respect to age, sex, or socio-economic status, which did not differ between overachievers and underachievers.”

In addition, we have now added Table 3 to describe these variables for the 3 group of voters.

Table 3. Socio-demographic characteristics of participants reporting voting for Mélenchon, Hamon or Macron (in the first round of the election) in our dataset.

| First-round vote | Age (SD) | Gender  (% women) | Occupation  (% White Collar) | Occupation  (% Student) |
| --- | --- | --- | --- | --- |
| Mélenchon | 31.21 (12.42) | 0.62 | 0.29 | 0.45 |
| Hamon | 32.57 (14.59) | 0.67 | 0.33 | 0.45 |
| Macron | 37.22 (16.75) | 0.45 | 0.47 | 0.32 |

Third, the findings may be biased by the fact that tasks are not incentivized as they would be in an ideal scenario. In particular, I worry that the lack of impact on most of the voters might be the consequence of the fact that the questions are asked hypothetically. A final question is raised as to whether one can believe that participants might have a desire to please who developed the study. However, there are not enough information on the structure of the survey, so it is very difficult to evaluate the validity of the research design.

Indeed, our measures are based on choices that were not incentivized. We have now added one paragraph in the discussion to highlight this difference between our study and the study of Deffains et al., and the role it may have played in our results.

“Our main result is that redistributive behavior is influenced by the exogenous manipulation of Status only in a subgroup of participants, specifically those who reported voting for Mélenchon. Therefore, our study partially replicated the findings of Deffains and colleagues (2016). This partial discrepancy between our study and that of Deffains might be due to incentives. In Deffains’ study, participants’ redistribution choices in the dictator game had real consequences on the payoffs of other players, whereas in our paradigm redistribution choices were only hypothetical. It has been proposed (e.g. Camerer & Hogarth, 1999) that in the absence of incentives, participants might try to please the experimenter or conform to some social norms, e.g. by being generous in dictator games. However, in our experiment, participants redistributed less than in Deffains’ study: our mean allocation to A was 60.08 while the corresponding value in Deffains’ study would be 57.56. Besides, it is not clear to us why this desirability bias would lead to the specific interaction between Status and First-round vote. Furthermore, since our experiment was conducted online and responses were anonymous, such an explanation in terms of desirability bias seems unlikely to us. Nevertheless, it is possible that incentives might have influenced our results independently of the desirability bias. Participants who reported voting for Hamon or Macron might be more sensitive to the presence of real life incentives than Mélenchon voters. Thus, incentivizing redistribution choices might be a necessary feature to obtain the effect of Status in Hamon or Macron voters, whereas Mélenchon voters would exhibit the effect of Status even in the absence of incentives. To evaluate these possibilities, further research would need to compare redistribution choices with and without incentives, for the different groups of voters.”
