# Supplementary material for: How does symbolic success affect redistribution in left-wing voters? A focus on the 2017 French presidential election
Source: PLoS One. 2020 Mar 16;15(3):e0229096. doi: 10.1371/journal.pone.0229096 (PMC7075674; doi:10.1371/journal.pone.0229096)
Supplement: Attachment — Submitted filename: Response to Reviewers.docx [file pone.0229096.s006.docx]

Reviewer #1: Referee’s report - Manuscript ID PONE-D-19-26429R2

Many thanks for the answers. However, I think that there is still something in need of clarification. In particular, my main concerns are about the exclusions of some observations and the balancing property of the sample. Please find below my comments to the previous answers.

REFEREE: First, the sample is heavily biased to left position and is far to be representative of the overall population. Although, I appreciate that authors are well aware about this limitation, I still think that this is a big issue. The worry is that one would expect the left-wing participants to possibly have quite different preferences from the general population. How much we can take from this work and extend to other groups or even other contexts?

AUTHOR/S: We are indeed aware of this limitation due to the bias in our sample, which was already acknowledged in the discussion. In the revised manuscript, we have made more explicit the possibility that right-wing or far-right voters could have exhibited a different behavior. Generalization to other populations and other contexts is always an issue in experimental studies. However, we believe that since it is an empirical issue, it can be tackled in future work.

REFEREE R2: Many thanks for your response. However, I`m still not sure about this point. The current title is a bit misleading. I would suggest you to make more explicit that your analysis focuses on left-wing voters. This also requires some additional effort in terms of framing the discussion. Sometimes, it seems that you are referring to the general population.

I also agree that representativity is always an issue in experimental studies. But, we run experiment to understand something more about preferences, behaviors etc. My main concern with this study refers to the fact that the sample is totally biased to the left and in particular to the “French left” during the time of the 2016 election … So, what can I learn from it? What in terms of value added to the existing literature? This something that should be explained better in the paper.

AUTHOR/S R2: We changed the title, and made our focus on left-wing voters more explicit in the abstract and in the main text.

In terms of representativity / general interest of our results, we now mention in the discussion that:

“We note that although our results may not be representative of right-wing voters, one could envision that they would generalize for left-wing voters in some other countries. Indeed, in the last decade, western democracies have seen a polarization of opinions, with a crisis of the traditional parties and a rise of support for extreme populist parties. Examples of populist far-left parties are die Linke in Germany, Podemos in Spain, Siriza in Greece, La France Insoumise (Jean-Luc Mélenchon’s party) in France. According to Rooduijn and Akkerman (2017) these radical left parties have in common that “They do not focus on the ‘proletariat’, but glorify a more general category: the ‘good people’” , contrary to former communist parties and that ”they do not reject the system of liberal democracy as such, but only criticize the political and/or economic elites within that system”. Our results regarding the susceptibility of Mélenchon voters to status manipulation could thus be evaluated and replicated in other countries.”

Regarding how our study contributes to the existing literature, we now highlight that our general finding that Mélenchon voters displayed a different behavior than Hamon and Macron voters connects with recent findings showing that supporters of extreme political groups have different characteristics from those with more moderate views (e.g. Rooduijn, 2018; Hanel et al., 2019).

We have re-written the last paragraph in the discussion section to address this point:

“To conclude, our findings revealed that self-reported far-left voters turned out to be the more sensitive to the exogenous manipulation of symbolic success. This leads to three remarks. Firstly, we need further research to better understand to what extent, and in which groups, redistributive behavior can be manipulated through exogenous manipulations of experience of success. In particular, further studies are needed that shall use a proper manipulation of symbolic success and representative samples in terms of political and socio-economic features. Secondly, our findings suggest that the various political groups process information differently, that is, they are not cognitively homogeneous (e.g., Amodio, Jost, Master, Yee, 2007; Rollwage, Dolan, & Fleming, 2018; Clarkson et al., 2015). Finally, and more broadly, the fact that Mélenchon voters displayed a different behavior than Hamon and Macron voters extends recent findings showing that supporters of extreme political groups have different characteristics from those with more moderate views, although they are not necessarily different on socio-demographic variables such as age or level of education (e.g. Rooduijn, 2018). For instance, Hanel, Zarzeczna, and Haddock (2019) reported that extreme (left-wing or right-wing) supporters are usually more heterogeneous than moderate ones in terms of human values and politics-related variables such as attitudes toward immigrants and trust in institutions. In the current social and political context, we believe that understanding further these differences, especially whether some groups are more susceptible to influence than others, appears a worthwhile subject for future research. Using controlled experiments during political elections can be a useful tool in such research.”

The added references are:

Rooduijn, M., & Akkerman, T. (2017). Flank attacks: Populism and left-right radicalism in Western Europe. Party Politics, 23(3), 193-204.

Rooduijn, M. (2018). What unites the voter bases of populist parties? Comparing the electorates of 15 populist parties. European Political Science Review, 10(3), 351-368.

Hanel, P. H. P., Zarzeczna, N., & Haddock, G. (2019). Sharing the same political ideology yet endorsing different values: left- and right-wing political supporters are more heterogeneous than moderates. Social Psychological and Personality Science, 10, 874-882.

REFEREE: Second, there are not enough information about groups characteristics. Are participants in the overachiever or underachiever groups different in their characteristics? Did the exclusions of some observations affect the balancing properties of the sample? How much people voting Hamon are different from those voting Macron, for example? These are very important information to understand the validity of the final results.

AUTHOR/S: We thank the reviewer for this comment. We have clarified in the discussion that group characteristics did not differ between Mélenchon voters and Hamon voters (Macron voters were older and more male on average), and between overachievers and underachievers.

REFEREE R2: I think that some answers are missing. I would suggest to address these points in the next version of the paper. Was the randomization before or after the exclusion of the observations? In my understanding, the randomization was before that some observations were excluded. Did the exclusion of these observations affect the balancing properties of the sample? It is always not good to exclude observations. If you do that, you should show that this will not affect your final results. My main concern is still: Are participants in the overachiever or underachiever groups different in their characteristics? author/s should show a table with these statistics and not only reporting two lines saying … “In addition, we verified that our Status manipulation was truly random with respect to age, sex, or socio-economic status, which did not differ between overachievers and underachievers.”

AUTHOR/S R2: We have updated Table 3 and added Table 4 to provide more information about our groups as well as the statistics for the comparison between overachiever and underachiever. We have moved these two tables up in the main text so they are now Table 1 and Table 2.

Table 1

Socio-demographic characteristics of participants reporting voting for Mélenchon, Hamon or Macron in the first round of the election in our dataset. Participants who reported another vote are pooled together in this table, and were not analyzed further in the present study.

| First-round vote | N | Age (SD) | Gender  (% women) | Occupation  (% White Collar) | Occupation  (% Student) | Status  (% overachiever) |
| --- | --- | --- | --- | --- | --- | --- |
| Mélenchon | 219 | 31.21 (12.42) | 0.62 | 0.29 | 0.45 | 0.46 |
| Hamon | 121 | 32.57 (14.59) | 0.67 | 0.33 | 0.45 | 0.57 |
| Macron | 166 | 37.22 (16.75) | 0.45 | 0.47 | 0.32 | 0.47 |
| Other | 120 | 32.40 (14.65) | 0.46 | 0.23 | 0.46 | 0.43 |

Table 2

Comparison of the socio-demographic characteristics of participants in the overachiever and underachiever conditions in our final sample (i.e. including only participants who reported voting for Macron, Hamon or Mélenchon).

| Condition | N | Age (SD) | Gender  (% women) | Occupation  (% White Collar) | Occupation  (% Student) |
| --- | --- | --- | --- | --- | --- |
| Overachiever | 248 | 33.73 (15.34) | 0.54 | 0.38 | 0.41 |
| Underachiever | 258 | 33.28 (14.06) | 0.60 | 0.34 | 0.40 |
| T |  | 0.344 | -1.371 | 0.888 | 0.187 |
| p |  | 0.731 | 0.171 | 0.375 | 0.852 |

REFEREE: Third, the findings may be biased by the fact that tasks are not incentivized as they would be in an ideal scenario. In particular, I worry that the lack of impact on most of the voters might be the consequence of the fact that the questions are asked hypothetically. A final question is raised as to whether one can believe that participants might have a desire to please who developed the study. However, there are not enough information on the structure of the survey, so it is very difficult to evaluate the validity of the research design.

AUTHOR/S: Indeed, our measures are based on choices that were not incentivized. We have now added one paragraph in the discussion to highlight this difference between our study and the study of Deffains et al., and the role it may have played in our results.

REFEREE R2: I`m fine with the discussion on the incentives but, again, the author/s did not reply to my final comment (or provide more information) about the potential desire of participants to please who developed the study.

AUTHOR/S R2: Our paragraph also addressed the issue of a potential desirability bias. Specifically, in our previous revision we wrote: “It has been proposed (e.g. Camerer & Hogarth, 1999) that in the absence of incentives, participants might try to please the experimenter or conform to some social norms, e.g. by being generous in dictator games. However, in our experiment, participants redistributed less than in Deffains’ study: our mean allocation to A was 60.08 while the corresponding value in Deffains’ study would be 57.56. Besides, it is not clear to us why this desirability bias would lead to the specific interaction between Status and First-round vote. Furthermore, since our experiment was conducted online and responses were anonymous, such an explanation in terms of desirability bias seems unlikely to us.”

We have now modified this passage to clarify this and better separate the discussion of incentives and the discussion of desirability bias. We now write in the discussion:

“It has been proposed (e.g. Camerer & Hogarth, 1999) that in the absence of incentives, participants might try to please the experimenter or conform to some social norms, e.g. by being generous in dictator games. Could this desirability bias explain our results or the difference between our study and Deffains’ study? We believe that such an explanation is unlikely for several reasons. First, if a desirability bias was more present our study than in Deffains’ study, then we should have observed more redistribution in our participants. However, in our experiment, participants redistributed less than in Deffains’ study: our mean allocation to A was 60.08 while the corresponding value in Deffains’ study would be 57.56. Second, and more generally, it is not clear to us why this desirability bias would lead to the specific interaction between Status and First-round vote. Third, the instructions given to participants (see Appendix A) did not refer to the aim of our experiment, so participants were naïve about our hypothesis. Had they tried to guess our expectations, we would have found an effect of status on fatalism, which we did not observe either in the full sample (p=.52) nor in Mélenchon voters (p=.35), whose redistributive behavior was affected by status however. Finally, our experiment was conducted online and responses were anonymous, so participants have no pressure to please the experimenter or conform to social norms.”
